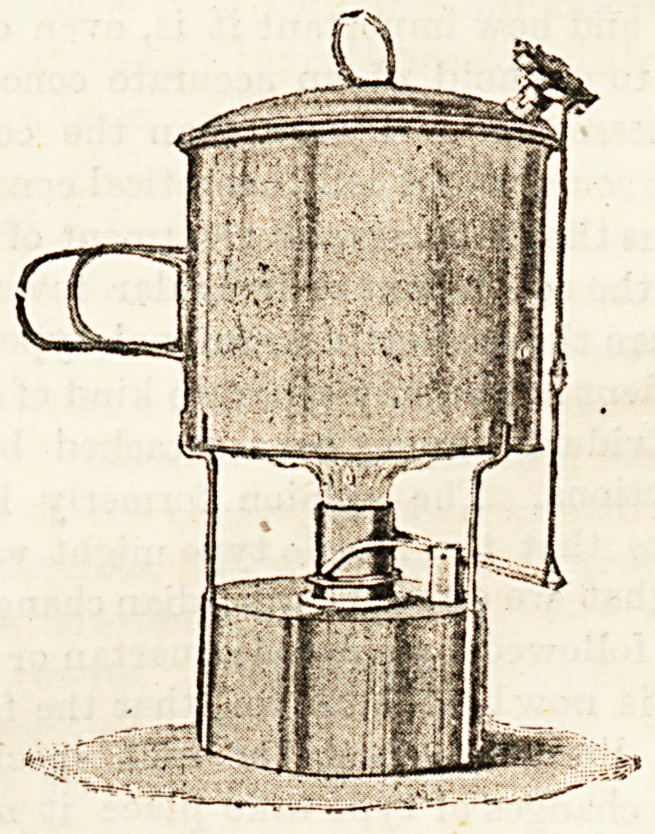# New Appliances and Things Medical

**Published:** 1900-08-25

**Authors:** 


### NEW APPLIANCES AND THINGS MEDICAL.

[We shall be glad to receive, at our Office, 28 & 29, Southampton Street, Strand, London, W.C., from the manufacturers, specimens of all new preparations and appliances which may be brought out from time to time.]

### THE SENTINEL PATENT STERILISER.

(The Cambridge Manufacturing Company, Limited, Llandaf Chambers, Regent Street, Cambridge.)

These sterilisers are automatic in action, and are adapted for use with gas, spirit lamp, or ordinary fire. They are so arranged that, when a certain temperature has been reached, the gas is turned off, the spirit lamp is put out, or a bell is rung. Although the principle can be so applied that any required temperature can be reached, in the case of these particular sterilisers the mechanism is "set" for a temperature of 95 deg. C. or 185 deg. F., a temperature which is found to be sufficiently high for the destruction of ordinary pathogenic bacteria. The advantages of such an arrangement are obvious; the milk cannot burn, neither can it boil over; the flavour and nutritive properties cannot be impaired by excessive boiling or high temperatures, and at the same time the milk is freed from living and dangerous micro-organisms. For nursery use the Sentinel Steriliser is a really practical and valuable contrivance, since it fulfils all the conditions which authorities insist upon as essential for rendering ordinary milk safe for infant consumption. For domestic and sick-room use its simplicity and reliability will find it many friends.

## Figures and Tables

**Figure f1:**